# Supplementary material for: Women's experiences and acceptability of self-administered, home delivered, intravaginal 5-Fluorouracil cream for cervical precancer treatment in Kenya
Source: Front Reprod Health. 2025 Feb 6;7:1487264. doi: 10.3389/frph.2025.1487264 (PMC11839715; doi:10.3389/frph.2025.1487264)
Supplement: Supplementary file 1 [file Table1.docx]

**Supplemental Table 1: Inclusion and exclusion criteria for the 5FU Phase I Pilot trial in Kisumu, Kenya**

| Inclusion Criteria | Exclusion Criteria |
| --- | --- |
| HIV-positive women (confirmed with serum antibody testing) | Unwilling or unable to use birth control during participation in the study |
| Age 18 years – 49 years at enrollment | History of invasive cervical cancer |
| Documentation of a biopsy-confirmed CIN2 or CIN3 | Known allergy to 5-Fluorouracil |
| Within 4-12 weeks after primary treatment (either cryotherapy, thermal ablation, or loop electrosurgical excision procedure (LEEP)) for CIN2 or CIN3 | History of total hysterectomy |
| Negative pregnancy test at screening | Presence of a condition or abnormality that in the opinion of the Investigator would compromise the safety of the patient |
| Agreement to use dual form of contraception (hormonal birth control, intrauterine device, or tubal ligation – plus condoms) during the study duration, if of childbearing age. Condoms can be male or female condoms | Current use of chemotherapeutic medication or high dose steroids (10 mg prednisone per day or more (or equivalent steroids) |
| Ability to understand and willingness to sign (or assent when applicable) informed consent |  |

**Supplemental Figure 1: Flow diagram of participant recruitment into the 5FU Phase 1 trial in Kisumu, Kenya**

Participants who underwent screening

(n) = 23

**Excluded (n) = 11**

Precancer treatment >12 weeks (n) = 5

Invasive disease (n) = 1

Mild dysplasia (CIN 1) = 1

Breastfeeding (n) = 1

Total hysterectomy (n) = 1

Other conditions (n) = 1 (symptomatic PID)

HIV non-disclosure to partner (n) = 1

Consented, screening procedures done, Enrolled (n) = 12

Completed 5FU doses & follow-up visits

n = 12

**Supplemental Table 2: Characteristics of women with cervical intraepithelial neoplasia grade 2/3 (CIN2/3) who participated in the 5FU Phase 1 trial in Kisumu, Kenya (n=12)**

| **Characteristic** | **n (%)** |
| --- | --- |
| **Age (years)** Mean (SD), Range (Min-Max) | 43.9 (4.4), 34-49 |
| **Highest education level attained** |  |
| Less than primary | 5 (41.7%) |
| Completed Primary | 2 (16.7%) |
| Completed Secondary | 4 (33.3%) |
| College or higher | 1 (8.3%) |
| **Occupation** |  |
| None | 3 (25.0%) |
| Salaried work | 3 (25.0%) |
| Business/Trader/Vendor | 2 (16.7%) |
| Farming | 4 (33.3%) |
| **Marital status** |  |
| Married/Living together | 5 (41.7%) |
| Divorced/Separated | 4 (33.3%) |
| Widowed | 3 (25.0%) |
| **Monthly income** |  |
| < Ksh 25,000 ($200) | 12 (100.0%) |
| **Electricity available** |  |
| Yes | 5 (41.7%) |
| **Tap water available** |  |
| Yes | 5 (41.7%) |
| **Current or prior tobacco use** |  |
| No | 12 (100.0%) |
| **Parity** Mean (SD) | 3 (2) |
| **Age at first sexual intercourse (years)** Mean (SD) | 17.2 (2.1) |
| **No. of lifetime sexual partners** Mean (SD) | 4 (2) |
| **CD4 count** |  |
| Median (Q1, Q3) | 781.0 (418.0, 890.5) |
| **Currently contraception use** |  |
| Yes | 6 (50.0%) |
| No | 6 (50.0%) |
| **Time since HIV diagnosis (Years)** |  |
| 1-5 years | 2 (16.7%) |
| > 10 years | 10 (83.3%) |
| **Currently on ARVs** |  |
| Yes | 12 (100.0%) |
| **No. of years on ARVs** |  |
| 1 - 2 years | 1 (8.3%) |
| Greater than 2 years | 11 (91.7%) |
| **Lifetime cervical cancer screenings** |  |
| One | 7 (58.3%) |
| 2-3 | 3 (25.0%) |
| More than 3 | 2 (16.7%) |
| **Prior cervical precancer treatments** |  |
| One | 12 (100.0%) |
| **Precancer results prior to recent treatment** |  |
| CIN 2 | 2 (16.7%) |
| CIN 3 | 10 (83.3%) |
| **Precancer treatment received** |  |
| Thermal ablation | 5 (41.7%) |
| LEEP | 6 (50.0%) |
| Cryotherapy | 1 (8.3%) |
